# 3D image scanning of gravel soil using in-situ X-ray computed tomography

**DOI:** 10.1038/s41598-023-46772-y

**Published:** 2023-11-16

**Authors:** Satoshi Matsumura, Akihiko Kondo, Keita Nakamura, Takaaki Mizutani, Eiji Kohama, Kenji Wada, Taizo Kobayashi, Nimisha Roy, J. David Frost

**Affiliations:** 1 Geotechnical Engineering Division, Port and Airport Research Institute, National Institute of Maritime, Port and Aviation Technology, 3-1-1 Nagase, Yokosuka, Kanagawa 239-0826 Japan; 2 Earthquake and Structural Dynamics Group, Earthquake Disaster Prevention Engineering Division, Port and Airport Research Institute, National Institute of Maritime, Port and Aviation Technology, 3-1-1 Nagase, Yokosuka, Kanagawa 239-0826 Japan; 3 Tsukuba Technology Co., Ltd., 1-14-11 Sengen, Tsukuba, Ibaraki 305-0047 Japan; 4 Department of Civil and Environmental Engineering, College of Science and Engineering, Ritsumeikan University, 1-1-1 Noji-Higashi , Kusatsu, Shiga 525-8277 Japan; 5 College of Computing, Georgia Institute of Technology, #253, 801 Atlantic DR NW, Atlanta, GA 30332 USA; 6 School of Civil and Environmental Engineering, Georgia Institute of Technology, #2285, 790 Atlantic Drive, Atlanta, GA 30332 USA

**Keywords:** Civil engineering, Imaging techniques

## Abstract

A typical ground investigation for characterizing geotechnical properties of soil requires sampling soils to test in a laboratory. Laboratory X-ray computed tomography (CT) has been used to non-destructively observe soils and characterize their properties using image processing, numerical analysis, or three-dimensional (3D) printing techniques based on scanned images; however, if it becomes possible to scan the soils in the ground, it may enable the characterization without sampling them. In this study, an in-situ X-ray CT scanning system comprising a drilling machine with an integrated CT scanner was developed. A model test was conducted on gravel soil to verify if the equipment can drill and scan the soil underground. Moreover, image processing was performed on acquired 3D CT images to verify the image quality; the particle morphology (particle size and shape characteristics) was compared with the results obtained for projected particles captured in a two-dimensional (2D) manner by a digital camera. The equipment successfully drilled to a target depth of 800 mm, and the soil was scanned at depths of 700, 750, and 800 mm. Image processing results showed a reasonable agreement between the 3D and 2D particle morphology images, and confirmed the feasibility of the in-situ X-ray CT scanning system.

## Introduction

A typical ground investigation to characterize soil properties requires in-situ sampling, where the void ratio, water content, and sedimentary structure are intact. Numerous sampling methods for retrieving intact soil specimens have been proposed for different soil types; most of them involve penetration into the target ground through a cylindrical sampling tube^1^. The soil samples in the sampling tube are carefully transported to a laboratory while avoiding disturbances. Then they are extracted from the tube and tested using a method appropriate for the specific purpose. If intact soil samples cannot be sampled or the quantity is insufficient for some reason such as cost, location, and soil types, for which any sampling methods are not suitable, disturbed soil may be used to reconstitute the soil samples in the laboratory for further testing. Notably, although the sampled soil can be reconstituted at a target void ratio and water content, the original sedimentary structure cannot be recreated because it is inherently inhomogeneous. This implies that the experimental results for the reconstituted soil samples are inevitably devoid of the sedimentary structure, which is known to have a significant impact on soil properties^2–9^. Therefore, regardless of whether intact soil samples are obtained in the field, the observation, quantification, and evaluation of natural sedimentary structures are essential.

X-ray computed tomography (CT) is a widely used non-destructive technique for examining objects in medical and industrial applications, as well as in research^10, 11^. This technique is useful in geotechnical engineering for observing sedimentary structures or discontinuous planes, such as fissuring, in soil samples^12^; to quantify physical properties, including particle size and shape characteristics^13–16^; to investigate pore and contact fabrics^17, 18^; or to characterize mechanical properties such as shear responses^19, 20^ primarily using the discrete element method (DEM)^21–24^ or three-dimensional (3D) printing technology to replicate synthetic samples^25–30^ based on CT images. Such a new paradigm based on imaging, image processing, and computing properties is often referred to as “digital rock physics”^31, 32^. Thus, in the case of the research objects being soils or rocks, the application of imaging tools such as X-ray CT scanners and computing methods is evolving.

In the aforementioned previous studies, soil or rock samples were obtained in the field or reconstituted in a laboratory before image scanning. The main research focus has been on techniques of processing CT images^17–20, 31, 32^, methods of incorporating the results of image processing into their computation^21–24^, and laboratory tests of synthetic samples^25–30^. Moreover, there are no studies on methods of acquiring CT images in-situ, or particularly, methods of scanning the soil samples on-site, that is, in the ground. However, scanning intact samples underground may enable the analyses described earlier (image processing and computing) without sampling and transporting or reconstituting the soil samples. Accordingly, this may reduce the number of soil samples required or enable the acquisition of more data on the ground when the transfer of samples is difficult, and there may be less concern regarding the disturbance in the soil samples induced by transportation, extraction from the sampling tube, and testing.

**Figure 1:**
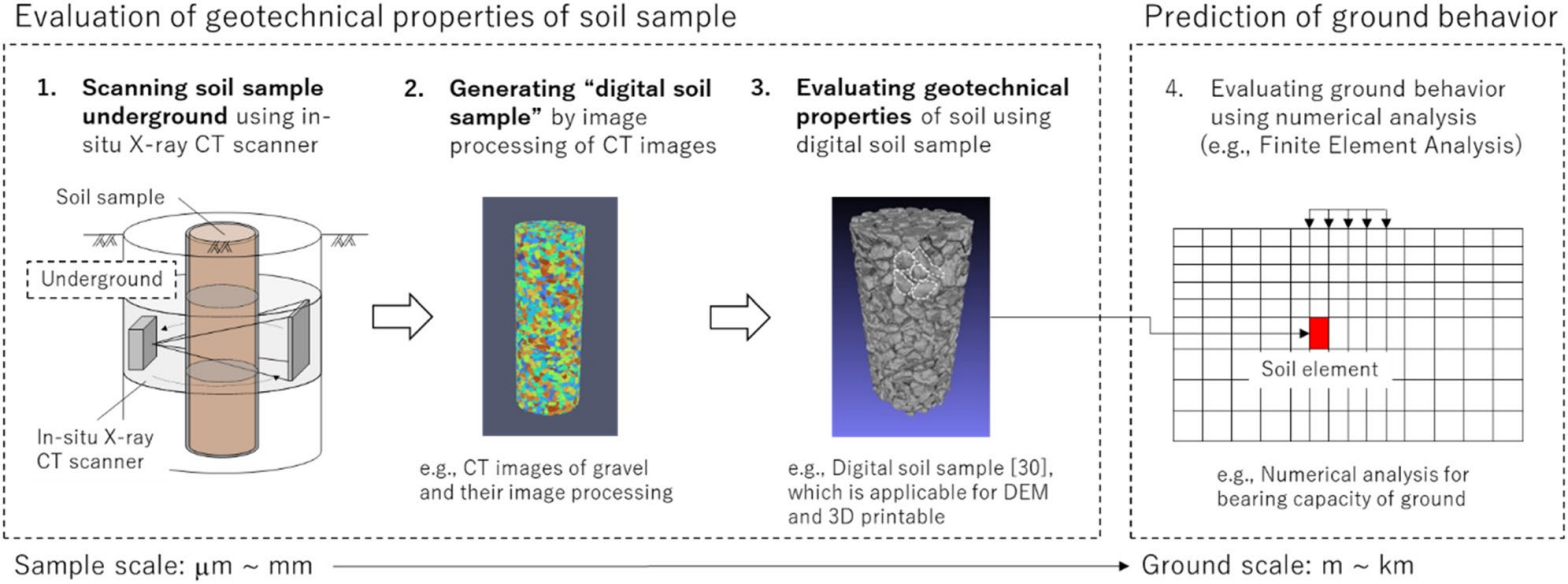
Concept of 3D-DIS and evaluation of geotechnical properties based on CT images.

Figure 1 illustrates the concept of X-ray CT for scanning soil samples underground (referred to as “3D digital image sampling” (3D-DIS) hereafter) to evaluate their geotechnical properties based on the CT images. The soil samples are scanned in-situ (i.e., underground), whereas in previous studies, they were sampled in-situ and scanned in a laboratory. Subsequent image processing provides CT images of soil samples that can be used to evaluate geotechnical properties; these can be described as “digital soil samples”. Using the digital soil sample, the geotechnical properties are evaluated through the image and numerical analyses, and/or 3D printing technique. Although these processes are implemented for the soil sample at a sample scale, ground behavior such as bearing capacity may be predicted at a ground scale using the sample-scale geotechnical properties. In this study, to investigate the feasibility of the 3D-DIS concept, an in-situ X-ray CT scanning system, i.e., self-drilling X-ray CT scanner (SeDX), was developed. SeDX primarily consists of a compact and portable X-ray CT scanner and a drilling machine into which the scanner can be installed. SeDX has the function of drilling and scanning the soil underground beside a sampling tube penetrated into the ground before SeDX drilling. A prototype system was tested in gravel soil to confirm whether it could drill the ground and scan the soil samples underground. Moreover, image processing was performed on the acquired images to verify whether the soil properties of the soil samples were appropriately captured. In particular, particle morphology (particle size and shape characteristics) such as the diameter of equivalent spheres and the length parameters of soil particles was investigated and compared with the results of a sieve test and image analysis of projected particles in captured two-dimensional (2D) images separately from the CT images. Notably, this study focuses on the feasibility of scanning the soil sample underground using SeDX and evaluating the quality of acquired CT images to generate the digital soil sample (Fig. 1); however, it does not include the evaluation of the geotechnical properties through a further image analysis, DEM, or 3D printing techniques.

The remainder of this paper is organized as follows: Section “Methods” describes basic concepts, configurations of the equipment developed for 3D-DIS, and the model test for verifying its feasibility; Section “Results” presents the model test results and image analysis of acquired CT images, followed by the challenges and prospects of the feasibility study and accomplishments; and finally, Section “Conclusions” presents the conclusions.

## Methods

### Design concept in developing equipment

To implement 3D-DIS using SeDX, two challenges must be addressed: the dimensions of the sample and equipment and the resolution of the target image. There is a trade-off affecting the equipment design, which must consider the target ground, image usage, and equipment specifications. For example, if the sample being examined is exceptionally big, the scanner must be large enough to capture it, thus requiring a higher extracted soil volume. In contrast, a small sample may lead to a reduction of drilling works and a higher resolution of the image, but this may be limited as a target sample zone; that is, it may not be suitable for soils composed of large particles like gravel. Eventually, air-dried and poorly-graded gravel soil was chosen as the target model ground. This is because the gravel soil’s individual particle shapes can be scanned and visualized with the specification of SeDX described in Section “Self-drilling X-ray CT scanner (SeDX)”, thus verifying the reliability of acquired images. Furthermore, the dried condition enables any waterproofing to be ignored, which considerably simplifies the equipment, model testing, and soil properties of the model ground.

Ultimately, the equipment was designed to satisfy three major requirements: (1) individual particle shapes of the gravel soil can be captured from scanned images using CT; (2) the soil sample to be scanned must be protected until it is scanned underground; and (3) the CT scanner must be as small as possible to minimize drilling requirements. SeDX (including the CT scanner and drilling machine) was designed based on these requirements. Moreover, to achieve the requirement (2), a sampling tube was necessary in addition to SeDX. The sampling tube, which is installed in SeDX, was penetrated into the ground to core and retain the soil sample for scanning before SeDX started to drill. Then, SeDX excavated the ground beside the sampling tube and advanced the CT scanner into the ground. The CT scanner, which can be installed inside SeDX, was used to scan the soil samples retained in the sampling tube.

### Self-drilling X-ray CT scanner (SeDX)

**Figure 2:**
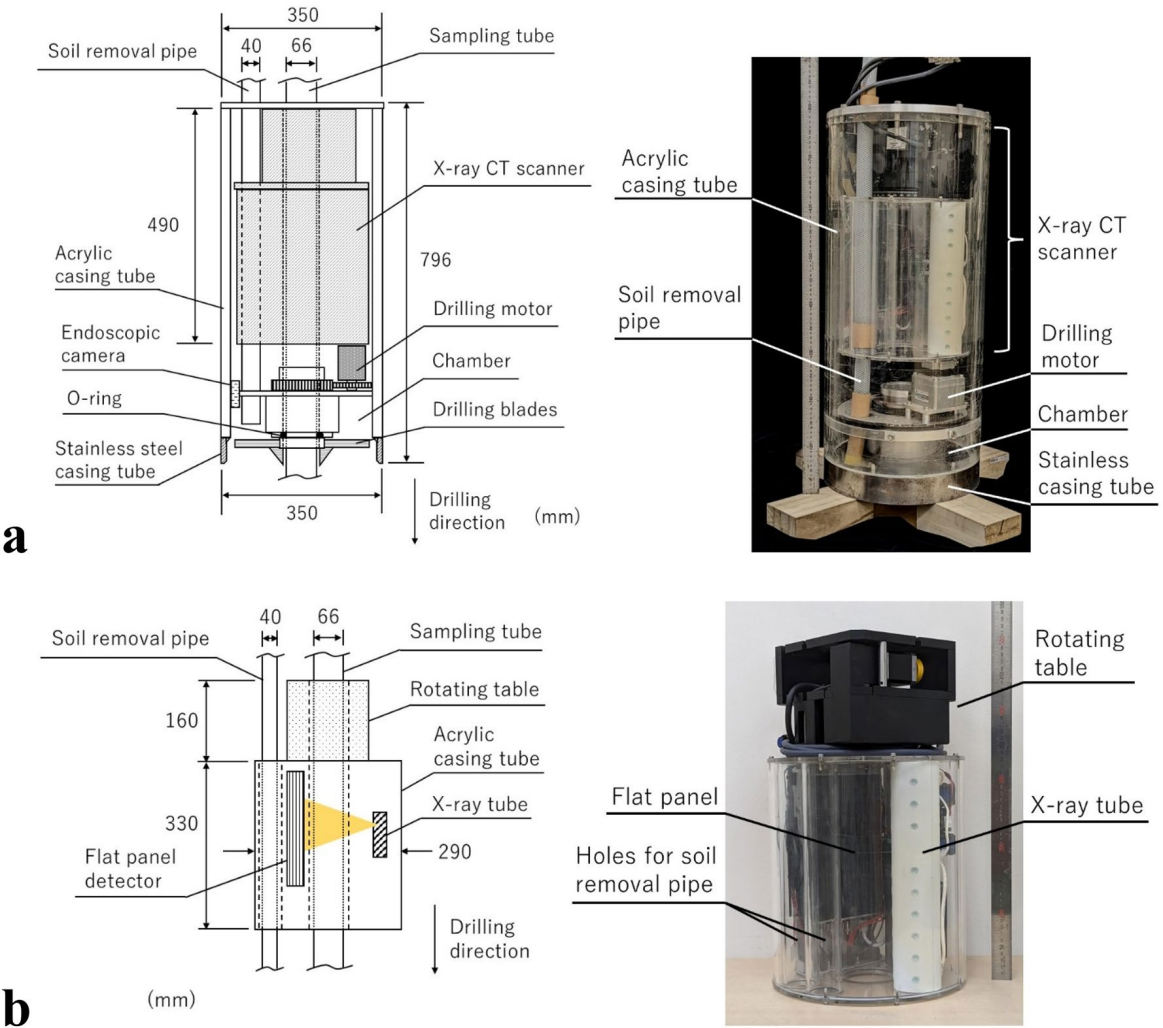
Basic equipment structure: (**a**) SeDX (left: schematic, right: picture) and (**b**) X-ray CT scanner (left: schematic, right: picture).

Figure 2a illustrates SeDX. The outside diameter and height of SeDX are 350 and 796 mm, respectively, and its design enables the scanner to be installed. SeDX excavates the soil using drill blades rotating at the tip and collects the excavated soil in a chamber. A vacuum machine (not shown in the figure) connected to the top of the soil-removal pipe withdraws the soil collected in the chamber. The inside of the chamber can be monitored using an endoscopic camera. When drilling, the body of SeDX moves straight ahead in the drilling direction without rotating. The soil reaction force during drilling is driven by the weight of SeDX and a drilling rig, which drives SeDX. This shield tunnel-like drilling method is considered essential for suppressing vibrations to avoid disturbing the soil sample and prevent the entire body from rotating. The casing tube is fabricated from an acrylic cylinder to enable the internal visual inspection of the devices and their movement. The tip, which is in contact with the soil ahead of the drill blades and receives the reaction force, is reinforced with stainless steel. The four straight drill blades are arranged orthogonally. SeDX has a 75-mm-diameter aperture at the center, through which the sampling tube with an outer diameter of 66 mm can pass.

As shown in Fig. 2b, the CT scanner, which is installed in SeDX, has a conical-beam X-ray tube, flat panel detector, and rotating table and includes a compact computer (not shown in the figure). These devices, excluding the rotating table, are covered by a dustproof donut-shaped acrylic casing. The CT scanner has a maximum external diameter of 290 mm and height of 490 mm and has two apertures for the sampling tube and soil-removal pipe. Table 1 lists the specifications of the CT scanner. The X-ray tube was originally developed by Kato et al.^33, 34^ and the dimensions and arrangements in the CT scanner were modified to the desired specifications. The maximum X-ray tube voltage and current were 120 kV and 1.0 mA, respectively. Noteworthy characteristics are its long-term stability at a high emission current and low power consumption^33, 34^. Moreover, because cold cathodes are used for the X-ray tube, no warm-up time before scanning is necessary. Additionally, the radioactive material is not used to generate X-rays; therefore, no residual radiation is left in the soil after the scanning process is completed. In field use, these properties could be advantageous in reducing the operating time and from an environmental perspective. Besides cost considerations, the flat panel detector was chosen according to the dimensions of the target soil sample and SeDX. The rotating table was custom-built and smaller than the external diameter of the CT scanner, with an aperture for the sampling tube.

**Table 1:** Main components of developed X-ray CT scanner and their specifications.

Items	Units	Names/Values
X-ray tube		
Model	–	Custom-built by Tsukuba Technology Co., Ltd
Tube voltage	kV	90–120
Tube current	mA	0.5–1.0
Irradiation time	sec	0.05–0.5
Flat panel detector		
Model	–	Dexela 1512GigE (Varex Imaging Corporation)
Size of detector	mm	115 × 145
Number of pixels	pixel	1536 × 1944
Size of a pixel	mm	0.0748
Depth of image	bit	16
Rotating table		
Model		Custom-built by SIGMAKOKI CO., LTD
Resolution of rotation	degree	0.1

### Other equipment: sampling tube and drilling rig

**Figure 3:**
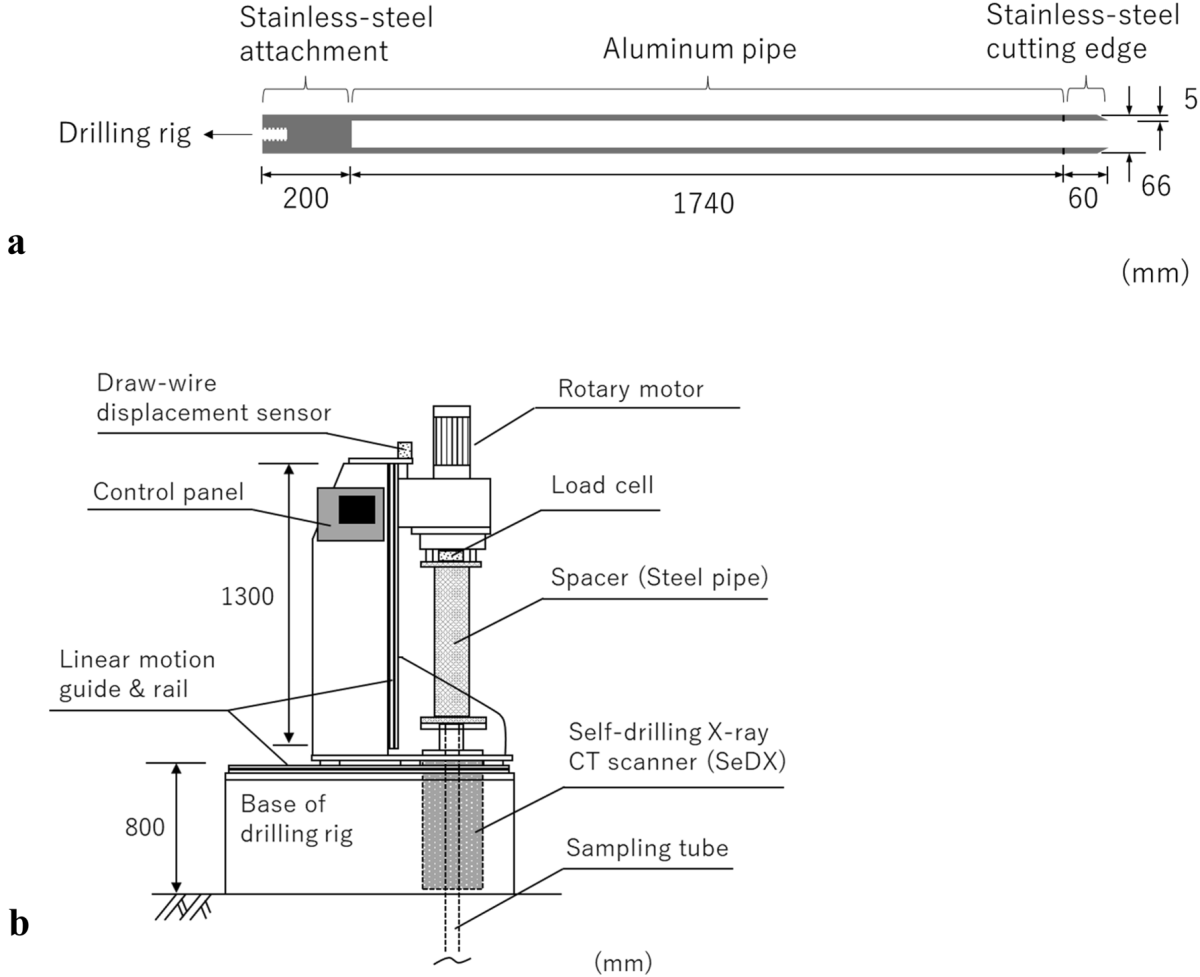
Basic equipment structure: (**a**) sampling tube and (**b**) drilling rig.

Figure 3a illustrates the sampling tube, and Table 2 lists its dimensions. The sampling tube consists of the stainless-steel attachment and cutting edge, and aluminum pipe. The outer and inner diameters of the aluminum pipe and stainless-steel cutting edge are 66 and 56 mm, respectively; accordingly, the thickness is 5 mm. The area ratio, which is the ratio of the area of the soil displaced by the sampling tube to that of the sample, is 38.9%. The inside clearance ratio, which is the ratio of the inner diameter of the sampling tube to that of the cutting edge, is 0%. Therefore, the sampling tube is categorized as a thick-walled sampler, which is recommended for sampling soils containing coarse particles such as gravel^1^. The total length of the sampling tube is 2000 mm. The stainless-steel cutting edge is formed with an angle tapered to 30°. The main body of the sampling tube is fabricated from aluminum piping and not steel, which is generally used. The unit weight of the aluminum pipe is sufficiently low to allow X-ray transmission, and the soil sample can be scanned while retained inside the sampling tube. The sampling tube can be passed through the center of SeDX holding the scanner and is in contact with a low-frictional O-ring made of Teflon that prevents decentering (Fig. 2a). Generally, it is difficult to core cohesionless gravel soil samples without disturbing the sedimentary structure even by a special sampling method for gravel such as a method using a thick polymer gel or solution as the drilling fluid^35^ or in-situ ground freezing sampling^6, 36^, because large gravel particles may move when the sampling tube penetrates the ground; this deteriorates the sample quality. The designed sampling tube was considered to disturb the soil samples to be scanned during the coring process; however, it was selected because this study aimed to verify if SeDX can drill the ground and scan the soil samples underground, even though the soil samples may be disturbed.

**Table 2:** Dimensions of the sampling tube.

Items	Symbols	Units	Values
Inner diameter of the cutting edge	*D*_1_	mm	56
Greatest outer diameter of the cutting edge	*D*_2_	mm	66
Inner diameter of the aluminum pipe	*D*_3_	mm	56
Outer diameter of the aluminum pipe	*D*_4_	mm	66
Tapered angle of the cutting edge	–	°	30
Length^a^	–	mm	2000
Area ratio^b^	*C*_a_	%	38.9
Inside clearance ratio^c^	*C*_i_	%	0

^a^Including the stainless-steel attachment and cutting edge and aluminum pipe illustrated in Fig. 3a.

^b^The ratio of the area of soil displaced by the sampling tube to that of the sample, defined by $\frac{{\left( {D_{2}^{2} - D_{1}^{2} } \right)}}{{D_{1}^{2} }} \cdot 100$^1^.

^c^The ratio of the inner diameter of the sampling tube to that of the cutting edge, defined by $\frac{{\left( {D_{3} - D_{1} } \right)}}{{D_{1} }} \cdot 100$^1^.

Figure 3b shows the drilling rig, which is used to drive SeDX. Moreover, it can be used to rotate the sampling tube at a maximum rotation velocity of 1600 rpm during penetration. The maximum length of the vertical displacement stroke is 1300 mm. As shown, after the sampling tube has cored the soil sample, a steel-pipe spacer is installed to connect the drilling rig to SeDX. The vertical displacement of the sampling tube and body of SeDX is monitored by a draw-wire displacement sensor located at the top of the drilling rig, while the vertical load of the SeDX is monitored by a load cell installed on the spacer.

### Model test ground

**Figure 4:**
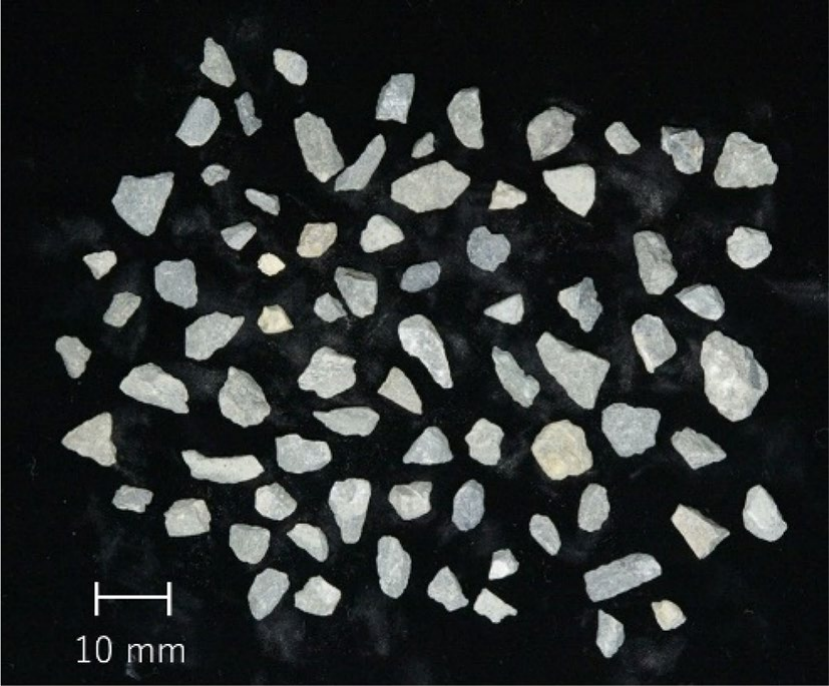
Gravel soil for testing the model.

Figure 4 shows the gravel soil used for testing the model prototype. Table 3 lists the soil properties of the gravel soil. The grain size distribution (GSD) was analyzed using sieves with sieve openings of 9.5, 4.75, and 2.0 mm based on the Japanese Industry Standard (JIS), JIS A 1204 “The method for particle size distribution of soils”. The maximum and mean grain sizes are 9.5 and 4.1 mm, respectively. The gravel content, in which the grains are larger than 2.0 mm, is 99.6%. The model ground was prepared by pouring the air-dried gravel soil to a depth of 1.1 m into a 1.0 m × 1.0 m wide × 1.3 m high container. The global dry density, which was measured using the total mass and volume of the model ground, was 1309 kg/m^3^. However, the model ground was repeatedly used for preliminary trial drilling tests, which somewhat disturbed the soil and crushed constituent particles. Thus, the local dry density and GSD probably differed from the global ones. However, because an accurate dry density and original GSD of the soil sample in the sampling tube was required to verify the scanned images, the dry density was measured through the testing process using the fresh gravel soil described in the next section.

**Table 3:** Properties of gravel soil.

Items	Symbols	Units	Values
Soil particle density^a^	*ρ*_s_	kg/m^3^	2727
Maximum dry density^b^	*ρ*_dmax_	kg/m^3^	1660
Minimum dry density^b^	*ρ*_dmin_	kg/m^3^	1275
Grain size^c^			
Percent finer than 9.5 mm	–	%	100
Percent finer than 4.75 mm	–	%	59.8
Percent finer than 2.0 mm	–	%	0.4
Maximum grain size^d^	*D*_max_	mm	9.5
Mean grain size	*D*_50_	mm	4.1
Maximum shear stress^e^	*q*_max_	kN/m^2^	271.0
Secant friction angle^e^	*ϕ*_0_	degree	46.9

^a^From JIS A 1202 “Test method for density of soil particles”.

^b^From the Japanese Geotechnical Society (JGS) standard, JGS 0162 “Test method for minimum and maximum densities of gravels”.

^c^From JIS A 1204, The method for particle size distribution of soils.

^d^Equal to maximum sieve openings.

^e^From JGS 0524 “Method for consolidated-drained triaxial compression test on soils”. Based on a consolidated drained triaxial compression test with a dry density of 1514 kg/m^3^ and confining stress of 50 kPa.

### Testing processes

**Figure 5:**
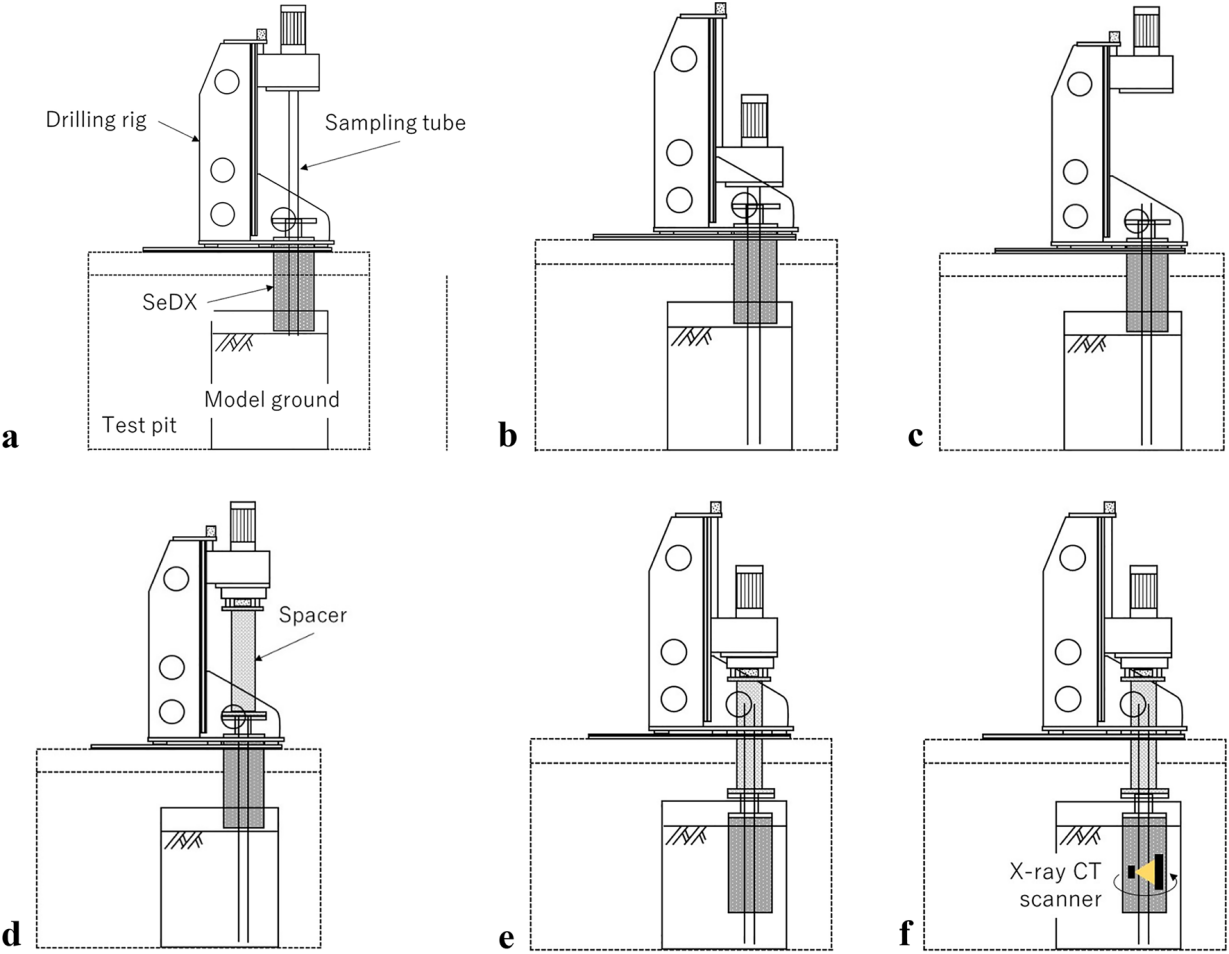
Stages for 3D-DIS using SeDX: (**a**) set-up of every component; (**b**) penetration of the sampling tube; (**c**) disconnection of the sampling tube from the drilling rig; (**d**) installation of a spacer to connect SeDX and drilling rig; € excavation of SeDX; and (**f**) X-ray CT scanning.

Figure 5 presents typical coring, drilling, and scanning processes using SeDX, the sampling tube, and drilling rig. Figure 5a shows the set-up of the equipment on the test ground. First, the sampling tube penetrates the ground to a given depth to retain the soil sample to be scanned before the drill (Fig. 5b). Subsequently, the sampling tube is disconnected from the drilling rig (Fig. 5c), while the spacer is placed between SeDX and the drilling rig (Fig. 5d). Next, SeDX drills the ground to the depth where the soil sample can be scanned (Fig. 5e). The scanner in the SeDX captures the image of the soil sample inside the sampling tube (Fig. 5f). After the scan, the processes illustrated in Fig. 5e and f can be repeated for subsequent scans. Notably, drilling pauses when the soil sample is scanned.

**Figure 6:**
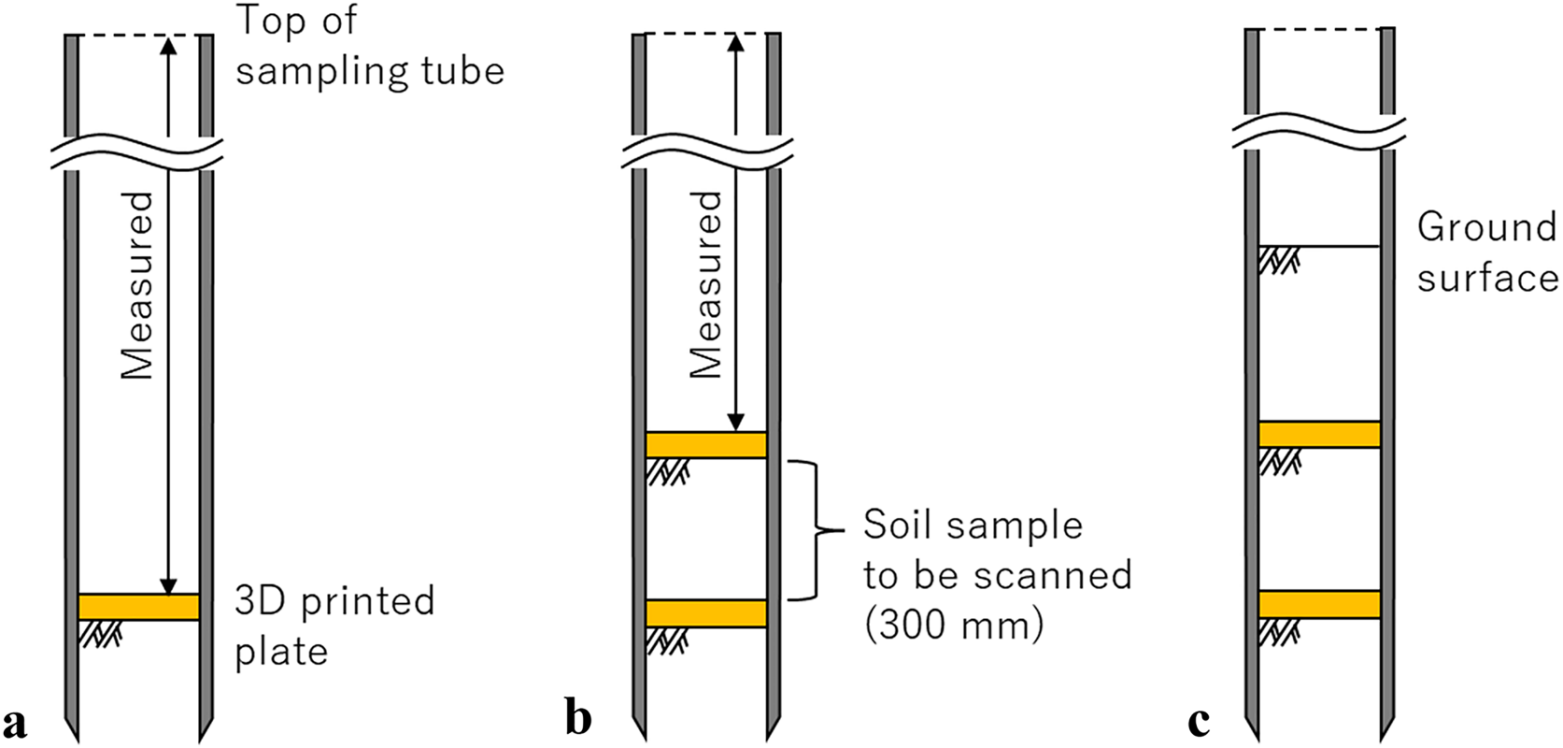
Additional processes for the preparation of the soil sample to be scanned.

Although this describes a typical process, in the model test, to assure the accurate dry density and original GSD of scanned soil sample for verification through image analysis, additional processes were performed between the processes shown in Fig. 5c and d, as illustrated in Fig. 6. Soils collected in the sampling tube during the process shown in Fig. 5c were removed up to the depth for nearly the bottom of a scanned soil sample. A 3D-printed plate with a diameter slightly smaller than that of the sampling tube and a thickness of 5 mm, was placed on the soil left in the sampling tube. The depth of the 3D-printed plate from the top of the sampling tube was measured (Fig. 6a). Subsequently, the sampling tube was refilled with a quantity of fresh gravel soil. Another 3D-printed plate with the same dimensions was placed on the refilled soil, and its depth from the top of the sampling tube was measured (Fig. 6b). The distance between the two 3D-printed plates was approximately 300 mm. The dry density of the soil sample to be scanned could be accurately analyzed using the refilled soil mass and volume, which was 1413 kg/m^3^. The relative density (*D*_r_) of the soil sample became 42%, which is considered medium dense. Finally, the sampling tube was refilled with the same soil up to the model ground surface. The processes were required because it is difficult to lift up the sampling tube while retaining the soil sample on the surface to measure the accurate dry density after the test owing to the absence of cohesion of the gravel soil, unless the soil is frozen or grouted. Moreover, the model test ground was repeatedly used, which appeared to disturb the soil with changing the dry density and GSD due to particle crushing.

During the model test, the drilling advanced at a vertical velocity of 0.5 mm/sec with the drill blades rotating at 12 rpm. During drilling, the vertical load, vertical displacement, and vertical and horizontal accelerations of SeDX were monitored. Moreover, the current flowing to the motor that rotates the drill blades (Fig. 2a), which relates to the resistance loaded onto the drill blades during drilling, was also monitored to observe whether the drilling motor stopped owing to an unexpected excess reaction force from the ground. SeDX excavated to a maximum depth of 800 mm. The soil sample was scanned thrice at depths of 700, 750, and 800 mm. Scanning depth intervals of 50 mm were selected so that the scanned areas of the soil samples slightly overlapped, enabling a continuous soil sample image to be generated. The scan was conducted with an X-ray tube voltage of 120 kV and an X-ray tube current of 0.9 mA, with a projection per second and 360 projections per scan. The resulting voxel size was 0.052 mm.

### Image processing and analysis for particle size and shape characteristics

The raw images captured in the prototype model test, that is, radiographs, were reconstituted to generate CT images using ConeCTExpress provided by ComscanTecno Co., Ltd. The CT images were processed and analyzed using OpenCV-Python version 4.6.0.66^37^ and the open-source Software for the Practical Analysis of Materials (SPAM) version 1^38^. The objective of the image analysis was to verify the quality of images against the physically measured geotechnical properties of the soil sample as well as properties measured using 2D projected images of particles captured by a digital camera (Fig. 4). Image processing was performed to: (i) remove noise; (ii) partition the image content into solids and voids; (iii) stitch the images acquired at different depths to generate a continuous soil sample image; (iv) segment individual particles; and (v) compute the particle size and shape characteristics. Furthermore, the properties calculated in (v) were compared to those calculated from 2D projected images of particles, which is described as processing step (vi).

(i) Noise removal

Scattering salt-and-pepper and tree-ring-like noise, termed ring artifacts, can appear in CT images, and degrade their quality. The relative total variation (RTV) was proposed as a useful image-processing method to remove these types of noise. RTV extracts a desired object, termed a “structure,” by removing an undesired pattern including scattering noise, which is termed “texture”^39^. This preserves the outlines of objects and differs from other typical denoising filters such as median or gaussian filters, which may produce blurring. Moreover, RTV removes the ring artifact in CT images^40, 41^. In the methods of^40, 41^, RTV creates a texture image of the ring artifact and eliminates the ring artifact by subtracting the texture image from the original image. If the first processing cycle does not remove the ring-artifact as desired, the creation of the texture image and subtraction can be repeated for the resulting images.

**Figure 7:**
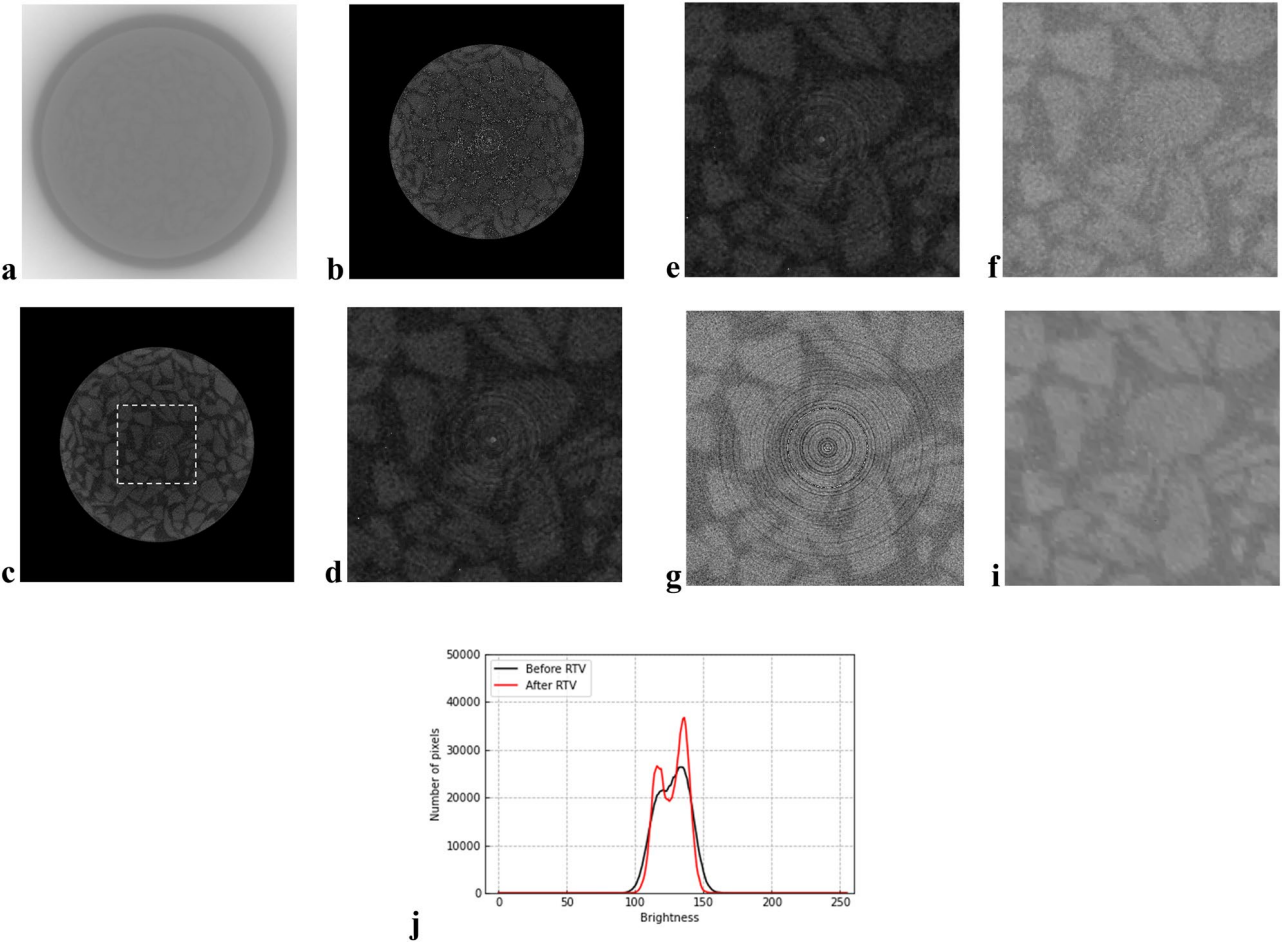
Effects of noise removal: (**a**) original horizontal slice image; (**b**) brightness adjustment; (**c**) median filter; (**d**) ring-artifacts remaining after median filtering (enlarged from Fig. 7c); (**e**) ring-artifact removal using RTV with one iteration; (**f**) ring-artifact removal using RTV with two iterations; (**g**) ring-artifact removal using RTV with two iterations without median filtering; (**i**) RTV after ring-artifact removal; and (**j**) comparison of the brightness distribution of images before and after RTV.

Figure 7 shows an original horizontal slice image acquired at a depth of 700 mm and its resulting images processed using the noise-removal method presented below. In this study, before noise removal, the brightness of horizontal slice images was adjusted to enhance their contrast based on a linear function presented by $\alpha x+\beta$, where x is the brightness before it was adjusted, whereas *α* and *β* are parameters to adjust the brightness. The function was selected because it is simple and does not lose any information of pixels, i.e., brightness. Parameters *α* and *β* were set as 10 and 10,000, respectively, by trial and error. The brightness-adjusted images were cropped to the soil sample area, i.e., the inner diameter of the sampling tube to eliminate an unnecessary domain (Fig. 7b). These steps were necessary for ease of visually assessing the image processing results. As shown in Fig. 7b, although solids (i.e., gravel particles) and voids can be visually confirmed, the scattering noise and ring artifacts are noticeable, deteriorating the image quality. Subsequently, the horizontal slice images were processed through the median filter with a kernel size of 5 × 5^37^. As described above, the median filter is useful for removing the scattering noise; however, the image may be somewhat blurred depending on the kernel size. Therefore, in this study, a relatively small kernel was employed. Figure 7c presents the resulting image processed through the median filter. The scattering noise was significantly reduced; however, the ring artifacts still remained, as shown in Fig. 7d, which is an enlarged image of the white-dotted domain in Fig. 7c. The median filter was used because the subsequent processing using RTV removed the noise more effectively. In this study, the images were converted from 16 to 8 bits to implement RTV, and based on parameter tuning, the parameters for RTV included λ, which controls the degree of smoothness and was set to 0.004, and σ, which specifies the maximum size of texture elements, sharpness, and iteration number^39^ was set to 4, 0.03, and 4, respectively. Figure 7e, f presents the results of ring-artifact removal processing using RTV^40, 41^. Two iterations that number the creation and subtraction of ring-artifact texture images as described above, were effective in removing the ring artifacts (Fig. 7f), whereas one iteration cannot remove them sufficiently (Fig. 7e). For comparison, Fig. 7g shows the resulting image processed in the ring-artifact removal method with two iterations, but without applying the median filter beforehand. Compared to the image shown in Fig. 7f, the ring artifacts deteriorated the image quality; therefore, the median filter was effective. However, Fig. 7f shows that the image appears to be still grainy. Therefore, after removing the ring artifacts, RTV was conducted to remove the remaining scattering noise with the same parameters. Consequently, RTV effectively removed the noise, resulting in clearer boundaries between the solids and voids (Fig. 7i). Figure 7j compares the brightness distribution of images before and after RTV processing, corresponding to Fig. 7f and i, respectively. Evidently, for the image after RTV processing, two clear peaks of brightness that indicate the solids and voids were confirmed, which appears easier to separate them in the subsequent processing step.

(ii) Partitioning the image content into solids and voids

**Figure 8:**
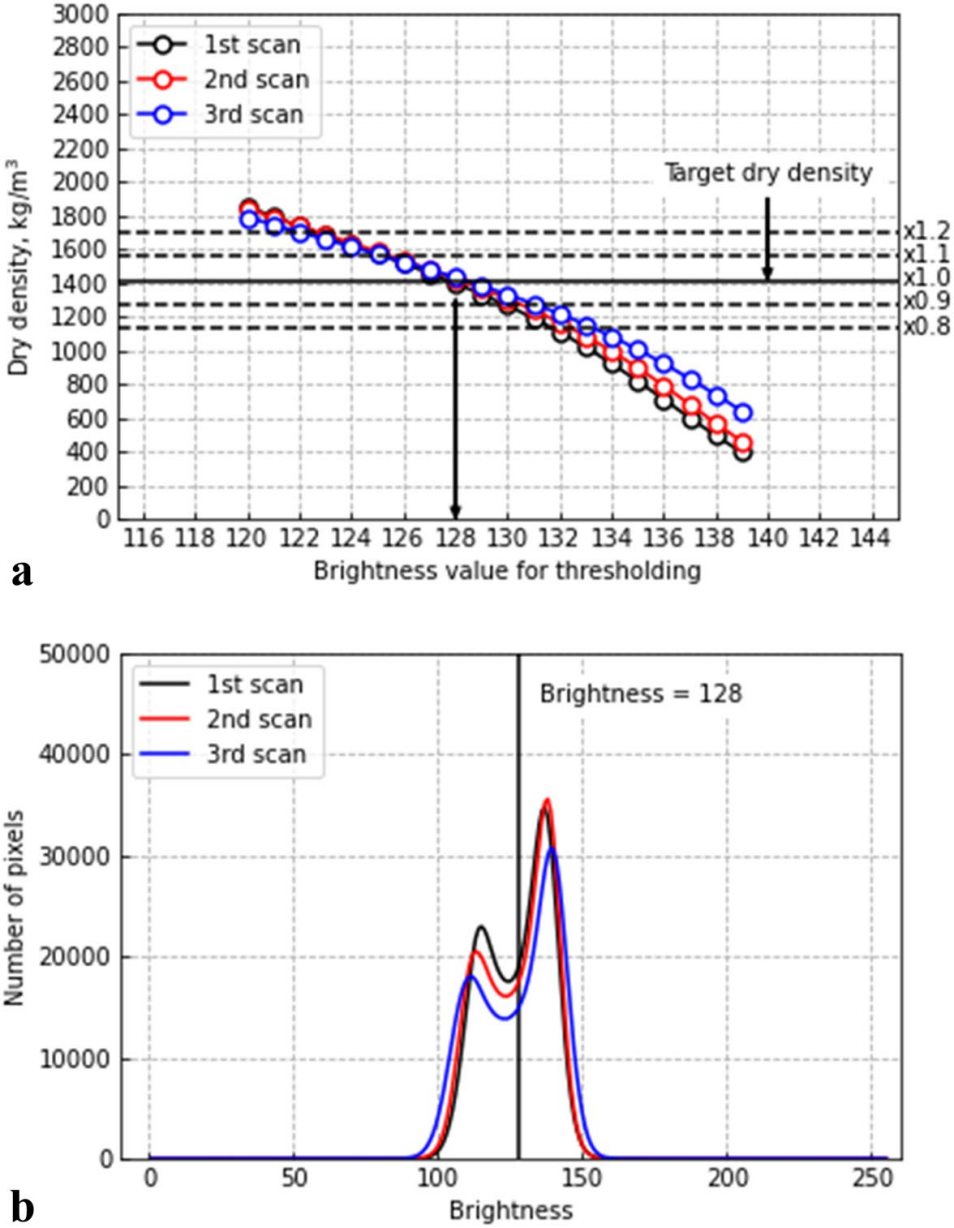
Effects of the threshold value: (**a**) relationship between the dry density and threshold value and (**b**) brightness distribution.

After denoising, the processed images were binarized to partition the image content into solid and void phases. The threshold for binarization was tuned for the dry density calculated from the volume of solid part and soil particle density (Table 3) to match the target dry density of the soil sample (1413 kg/m^3^) measured in the laboratory. This process was individually applied to three sets of CT images acquired at different depths of the sample. Figure 8a shows the relationship between the calculated dry density and threshold values. The threshold value was set to 128, which falls in the mid-range of the 8-bit pixel value spectrum that spans from 0 to 255. With the threshold value of 128, the calculated dry densities for the first, second, and third scans were 1395, 1423, and 1430 kN/m^3^, respectively, which correspond to 1416 kN/m^3^ on average. The dotted lines in the figure indicate that the brightness within ± 10 and ± 20% of the target dry density ranges from 126 to 130 and from 122 to 132, respectively. A slight difference of threshold value appears to significantly affect the calculated dry density; therefore, for the practical use of SeDX, a careful calibration of the threshold value to reference dry densities is required. Figure 8b presents the average brightness distribution for three sets of CT images scanned at different depths and processed through the denoising method. As well as the tendency shown in Fig. 7j, for every dataset, two clear peaks of brightness indicating the solids and voids appear, and the threshold value (i.e., 128) lies between the two peaks.

(iii) Stitching images acquired at different depths

**Figure 9:**
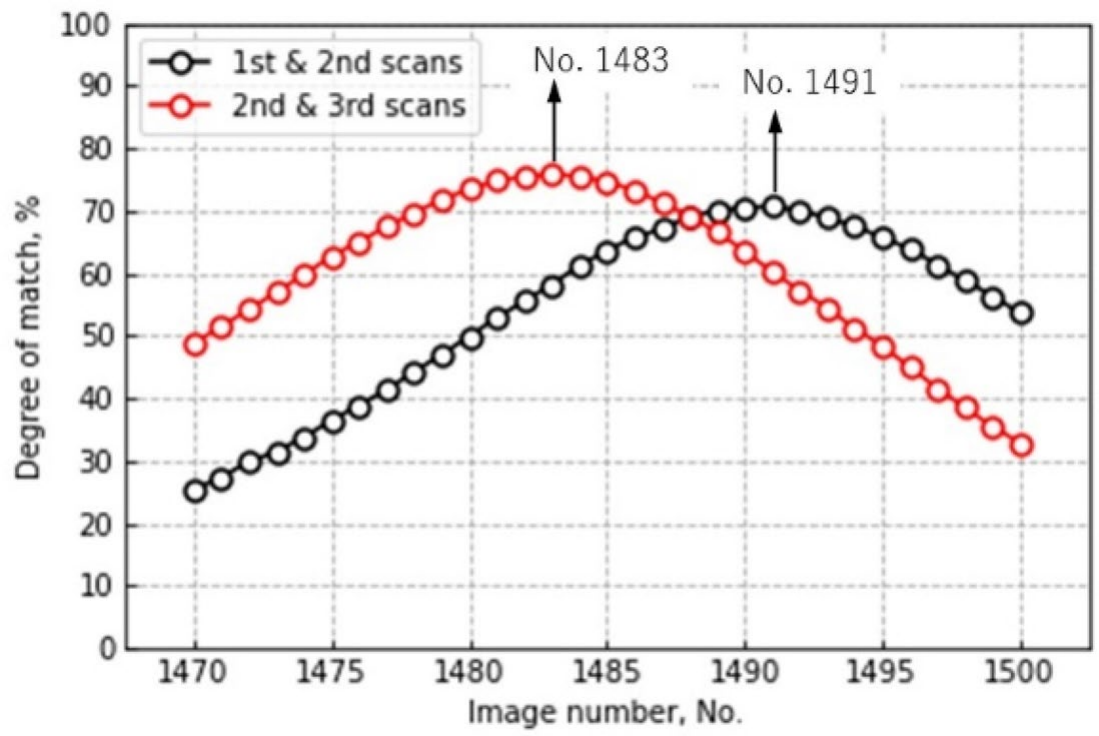
DOM expressed as a percentage against the image number.

To stitch the images appropriately, the same depth must be determined for different image datasets. The same depth was determined by the degree of match (DOM) defined as:

1 $$DOM=\sum {n}_{0}(x, y)/{n}_{t}$$

where n_t_ is the total number of pixels in an image and n_0_(x, y) is defined as:

2 $$n_{0} \left( {x, y} \right) = \left\{ {\begin{array}{*{20}c} {1\;if \left| {I_{1} \left( {x, y} \right) - I_{2} \left( {x, y} \right)} \right| = 0} \\ {0\;if \left| {I_{1} \left( {x, y} \right) - I_{2} \left( {x, y} \right)} \right| \ne 0} \\ \end{array} } \right.$$

where I_1_(x, y) and I_2_(x, y) are horizontal slice binary images composed of x × y pixels at the different datasets. Therefore, the differences between the two horizontal slice images in the previous and subsequent datasets were computed, and the pixels with zero brightness, i.e., n_0_(x, y), were counted. If DOM is the highest, the two horizontal slice images in the previous and subsequent datasets are located at the closest height. Each dataset contributed 1944 horizontal slice images. As shown later in Section “Image processing and analysis for particle size and shape characteristics”, in the vertical direction, the top of each image had a blind spot because of the placement of devices such as the X-ray tube and detector and could not be properly acquired. Therefore, the top 500 images were not used in this study. Figure 9 shows the DOM expressed as a percentage versus image number in the previous datasets. For example, the result indicates that the 1491st image in the 1st scan best matched the 501st image in the 2nd scan, whereas the 1483rd image in the 2nd scan best matched the 501st image in the 3rd scan.

(iv) Segmenting individual particles

Using SPAM functions^38^, the raw binary images were processed in three steps that included: (a) cleaning the image via morphological opening and closing operations to smoothen borders and fill holes; (b) labeling the cleaned image to segment individual particles; and (c) cleaning the image by fixing over- and under-segmentation based on the physical size of smallest and largest particles measured in the laboratory. More details regarding segmentation were reported by Roy et al.^20^.

(v) Computing the particle size and shape characteristics (for 3D images)

Because the particle morphology, i.e., particle size and shape characteristics, can influence the mechanical properties of granular material, various parameters that characterize them have been proposed and investigated^13, 14, 16, 42–53^. In this study, the particle morphology was evaluated using SPAM functions^38^ with the parameters listed in Table 4: the diameter of an equivalent sphere that has the same volume as a target particle (*D*_eq-3D_); lengths of the minor (*L*_s-3D_), intermediate (*L*_i-3D_), and major axes (*L*_l-3D_) of an ellipsoid fitted to the target particle; aspect ratios calculated by *L*_s-3D_/*L*_l-3D_ (*AR*_sl-3D_) and *L*_i-3D_/*L*_l-3D_ (*AR*_il-3D_); and true sphericity (*S*_t-3D_) calculated using *S*_eq_/*S* ^42^. *S*_eq_ denotes the surface area of a sphere with the same volume as the target particle, and *S* denotes the surface area of the particle. These size and shape descriptors were selected because they were often adopted to compare particle morphologies in 2D and 3D images^13, 14, 16, 46, 49^, even though there is no general agreement on the types of descriptors that are the most comparable. Regarding sphericity, various definitions for 2D and 3D images have been proposed^42, 45, 53^. In this study, *S*_t-3D_ and perimeter sphericity (*S*_p-2D_) were adopted in step (vi), which are the most comparable^14^.

**Table 4:** Particle size and shape characteristics for 3D images.

Items	Symbols	Computation methods
Diameter of an equivalent sphere	*D*_eq-3D_	(6* V*/π)^1/3a^
Length of an ellipsoid		
Minor axis	*L*_s-3D_	–
Intermediate axis	*L*_i-3D_	–
Major axis	*L*_l-3D_	–
Aspect ratio		
Minor axis vs. major axis	*AR*_sl-3D_	*L*_s-3D_/*L*_l-3D_
Intermediate axis vs. major axis	*AR*_il-3D_	*L*_i-3D_/*L*_l-3D_
True sphericity	*S*_t-3D_	*S*_eq_/*S*^b^

^a^*V* is the volume of the segmented particle.

^b^*S*_eq_ is the surface area of a sphere having the same volume as the target particle; *S* is the surface area of the particle.

Using the particle size descriptors, GSD was computed to compare it to that from the sieve analysis. For the ellipsoid-fitted particles, the percent finer was calculated depending on the length of each axis, which is *L*_s-3D_, *L*_i-3D_ or *L*_l-3D_; that is, in the case of *L*_s-3D_, for example, it was calculated assuming that only *L*_s-3D_ determines if the particle is smaller or larger than the sieve opening size. The mass of particle was calculated using the soil particle density (Table 3) and volume calculated using Eqs. (3) and (4) for the sphere- and ellipsoid-fitted particles, respectively.

3 $$\frac{4}{3}\pi \left( {\frac{{D_{eq - 3D} }}{2}} \right)^{3}$$

4 $$\frac{4}{3}\pi \left( {\frac{{L_{s - 3D} }}{2}} \right)\left( {\frac{{L_{i - 3D} }}{2}} \right)\left( {\frac{{L_{l - 3D} }}{2}} \right)$$

(vi) Computing the particle size and shape characteristics (for 2D images)

Images of 700 sample particles placed in stable directions distinct from adjacent particles were captured using a digital camera and analyzed using OpenCV-Python functions^37^. Figure 4 shows part of the analyzed sample particles. The resolution of the image was 0.032 mm/pixel. The image was binarized to separate particles and backgrounds. Subsequently, as listed in Table 5, for the segregated particles, the radius of a circle that had the same area as the target particle (*D*_eq-2D_), the lengths of the minor (*L*_s-2D_) and major axes (*L*_l-2D_) of an ellipse fitted to the target particle, the aspect ratio (*AR*_sl-2D_) calculated using *L*_s-2D_/*L*_l-2D_, and *S*_p-2D_ calculated using *P*_eq_/*P*^53^ were analyzed. *P*_eq_ denotes the perimeter of a circle having the same area as the target particle, and *P* denotes the perimeter of the particle.

**Table 5:** Particle size and shape characteristics for 2D images.

Items	Symbols	Computation methods
Diameter of an equivalent circle	*D*_eq-2D_	2(*A*/π)^1/2a^
Length of an ellipse		
Minor axis	*L*_s-2D_	–
Major axis	*L*_l-2D_	–
Aspect ratio		
Minor axis vs. major axis	*AR*_sl-2D_	*L*_s-2D_/*L*_l-2D_
Perimeter sphericity	*S*_p-2D_	*P*_eq_/*P*^b^

^a^*A* is the area of the projected particle.

^b^*P*_eq_ is the perimeter of a circle having the same area as the target particle; *P* is the perimeter of the particle.

## Results

### Drilling and scanning processes

**Figure 10:**
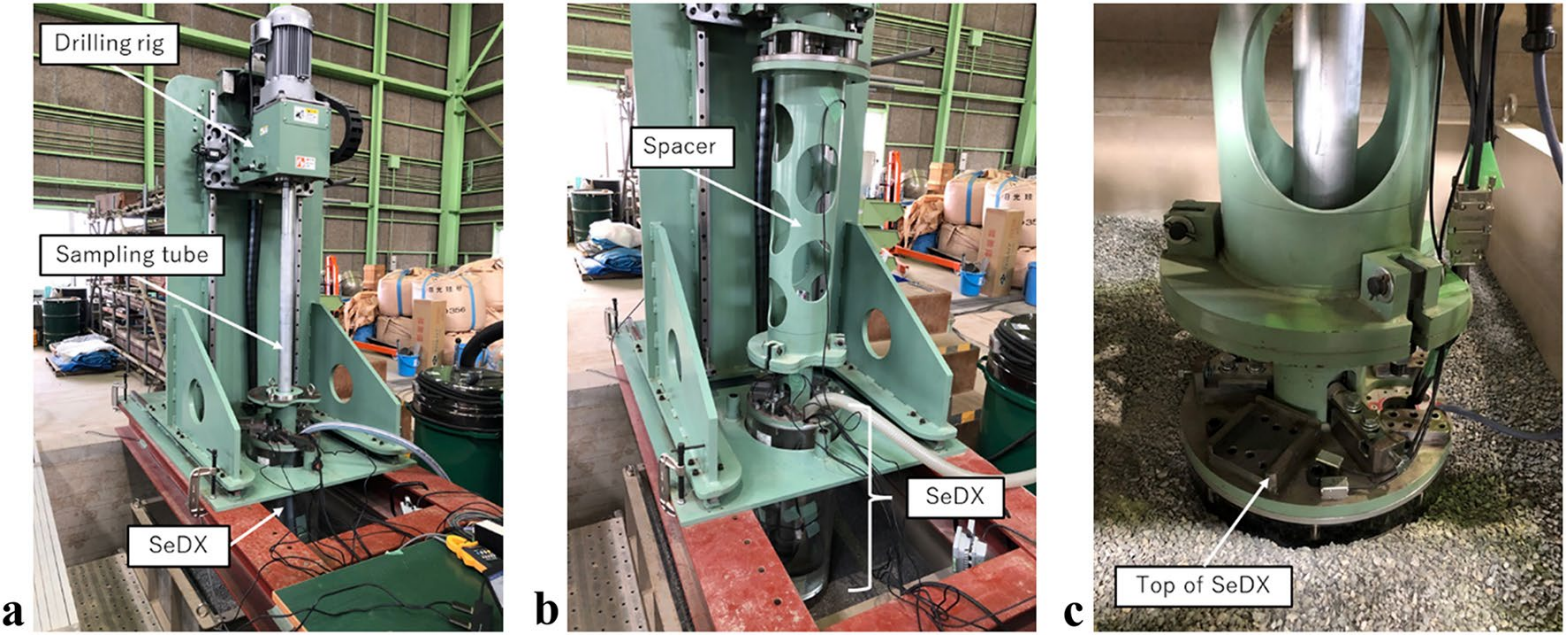
Model test: (**a**) set-up (corresponding to Fig. 5a); (**b**) before SeDX excavated the ground (corresponding to Fig. 5d); and (**c**) after SeDX excavated the ground (corresponding to Fig. 5e).

Figure 10 shows images captured while testing the prototype model. Figure 10a shows the set-up of equipment including SeDX, the sampling tube, and drilling rig, which corresponds to the process illustrated in Fig. 5a. The sampling tube was penetrated vertically at approximately 30 mm/sec without rotation up to a depth of 900 mm. Figure 10b shows SeDX after the sampling tube was penetrated and before the drilling process started, which was illustrated in Fig. 5d. Figure 10c shows the top of SeDX that finished excavation, which was shown in Fig. 5f. SeDX successfully excavated to a final depth of 800 mm, which was 100 mm shallower than the final depth of the sampling tube to retain the soil samples, and it paused at 700 and 750 mm to scan the soil sample.

**Figure 11:**
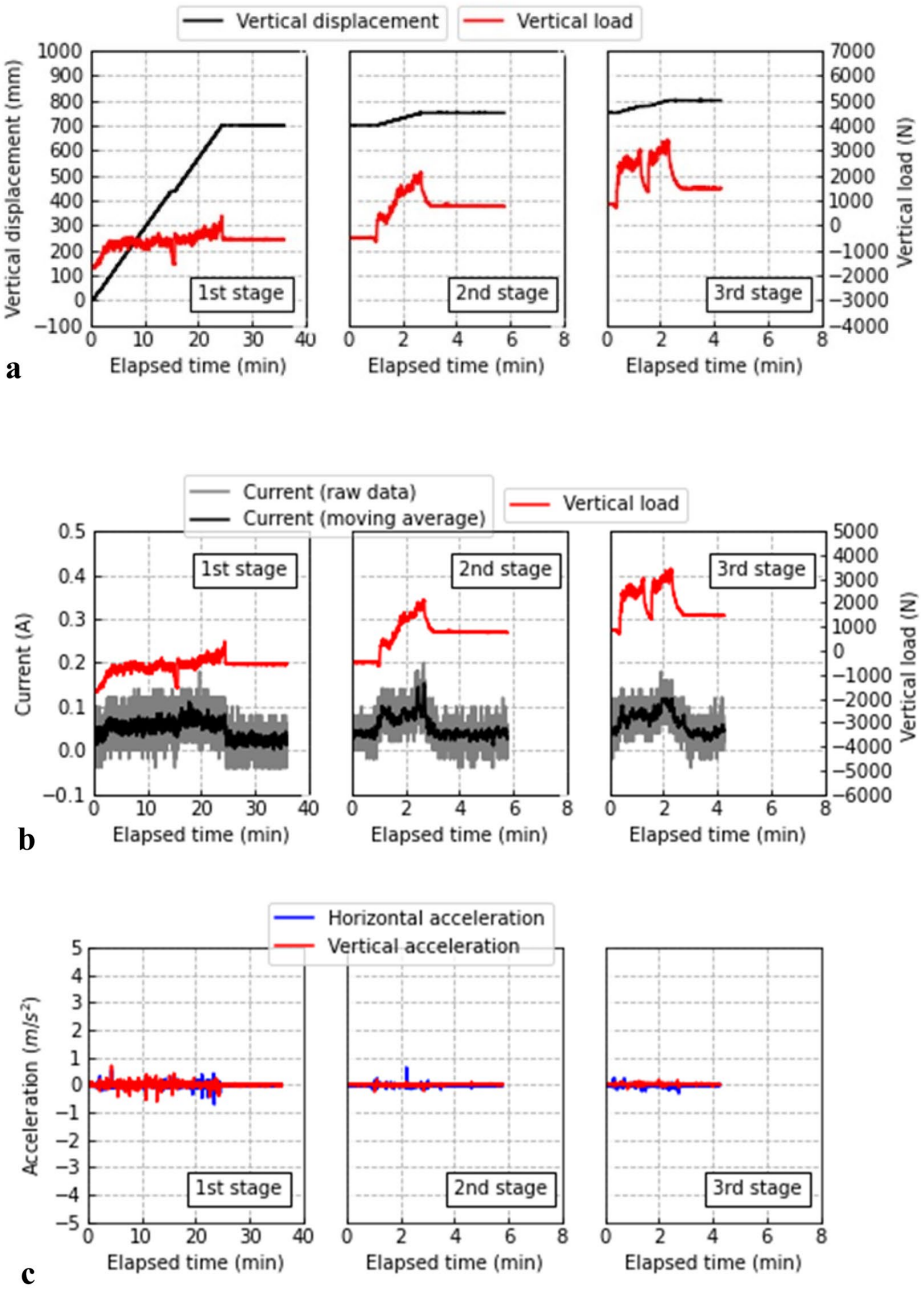
Results of drilling: (**a**) vertical displacement and force and (**b**) current and vertical force; (**c**) vertical and horizontal accelerations.

Figure 11 shows the time histories of the values monitored during drilling divided by the first, second, and third stages at depths of 0–700, 700–750, and 750–800 mm, respectively. The elapsed time of 0 s at the second and third stages corresponded to the time after each previous stage was completed. From Fig. 11a, the vertical load increased from approximately − 1700 N, which corresponds to the weight of SeDX. As shown, the vertical load continuously increased as the vertical displacement increased. Abrupt decreases at 15 min of the first stage and 1.5 min of the third stage were attributed to stress relaxation due to excavation being paused to remove soils collected in the chamber. Compared to the first stage, the vertical load drastically increased for the second and third stages. A possible reason is that the tip of SeDX approached the bottom of the model container, thus causing a higher vertical load due to the model container exerting a confining effect. As shown in Fig. 11b, the current appears to react to a change in the vertical load. This could be due to the drill blades enduring a vertical reaction force from the ground when rotating. However, in the prototype model test, the drilling process was successfully completed without any stoppage of the drill blades. Figure 11c shows that vertical and horizontal accelerations, which were monitored on the top of SeDX, remained within ± 1 m/s^2^ during drilling. Although the accelerations were monitored to reveal optimal drilling conditions and the permissible vibration to input to the X-ray CT scanner, it is also important to clarify the effects of the vibration on the soil sample. Thus far, the cyclic stress ratio (CSR) input to the ground during cyclic loading such as Earthquake, which is defined by Eq. (5), is considered^54^.

5 $$0.65 \cdot \frac{{\sigma_{z} }}{{\sigma_{z}^{\prime } }} \cdot \frac{{\alpha_{\max } }}{g} \cdot r_{d}$$

where *σ*_z_ and *σ*_z_^′^ are the total and effective vertical stresses, respectively; *α*_max_ is the maximum horizontal acceleration; g is the gravitational acceleration (9.8 m/s^2^); and *r*_d_ is the stress-reduction coefficient to reduce the cyclic stress depending on the depth. In this study, *σ*_z_ = *σ*_z_^′^ because dried soil was used. Moreover, a rough estimate of *α*_max_/g can be 0.1 (≈1/9.8) based on Fig. 11c. *r*_d_ $\approx$ 1 near the ground surface. Eventually, CSR applied on the soil sample is assumed as 0.065. Sassa et al.^55^ investigated the volume contraction of rock debris during cyclic shear loading. Their result clarified that the residual volume strain was lower than 1% under *σ*_z_ = 49 kPa and CSR = 0.21, which is approximately three times higher than 0.065, even for the rock debris with *D*_r_ = 35%, which was the most contractive for the tested soils and conditions. Therefore, in this study, the volumetric strain of soil samples in the sampling tube is assumed to be much lower than 1%. Moreover, unlike in the first stage, the accelerations for the second and third stages appear to decrease. This was probably because the vibration induced by drilling was reduced due to the higher earth pressures at greater depths in the ground. The initial *D*_r_ was 42%, when fresh gravel was refilled into the sampling tube, which is medium dense, not too loose. The sampling tube contacts SeDX at the low-frictional O-ring only, and the two are not rigidly connected. As shown in Fig. 10c, the ground surface beside SeDX appears to cause no significant settlement. Therefore, these experimental results indicate that the effects of the vibration during drilling appear to be insignificant to densifying the soil sample. Furthermore, as shown in Fig. 8a, the variation of dry density (i.e., volumetric strain) of less than 1% is insignificant to the threshold value; therefore, the volume change of the soil sample due to the vibration did not significantly affect the image analysis results.

### Image processing and analysis for particle size and shape characteristics

**Figure 12:**
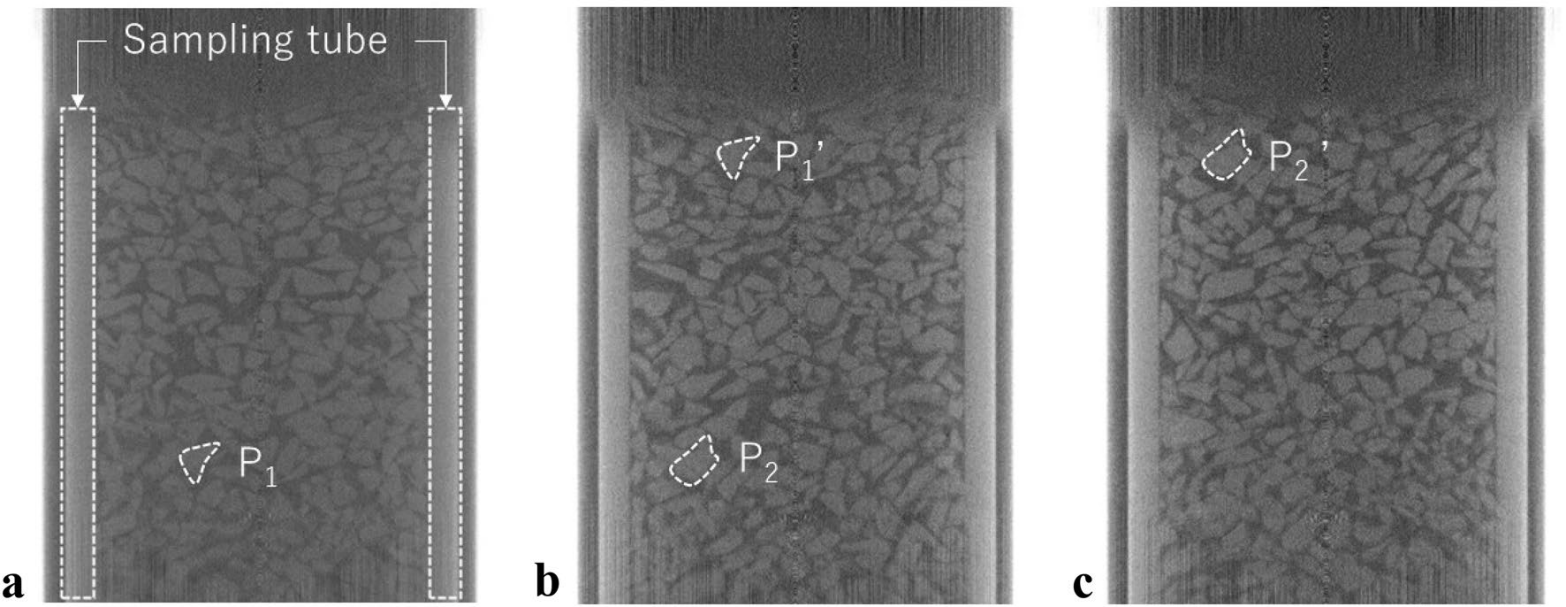
Vertical slice images (1536 × 1944 pixels) at the center of the soil sample; (**a**) first scan (at 700 mm) and (**b**) second scan (at 750 mm); and **c**. third scan (at 800 mm). Note that the brightness was adjusted for viewability.

Figure 12 shows the raw vertical slice images across the centers of the soil samples acquired at different depths: 700, 750, and 800 mm. In the images, the gray irregularly shaped objects correspond to particles, while the darker interparticle domains are air voids. The sizes and shapes of individual gravel particles and their sedimentary structures sampled in the aluminum sampling tube were successfully visualized. The particles were randomly deposited, and accordingly, interparticle voids of various sizes were also randomly generated. In Fig. 12, the particles with white-dotted outlines confirm that the soil sample was continuously scanned and that the scanned domains overlapped. Particle P_1_ shown in the first scan (Fig. 12a) was observed as P_1_’ in the second scan (Fig. 12); P_2_ in the second scan and P_2_’ in the third scan were also the same (Fig. 12b and c).

**Figure 13:**
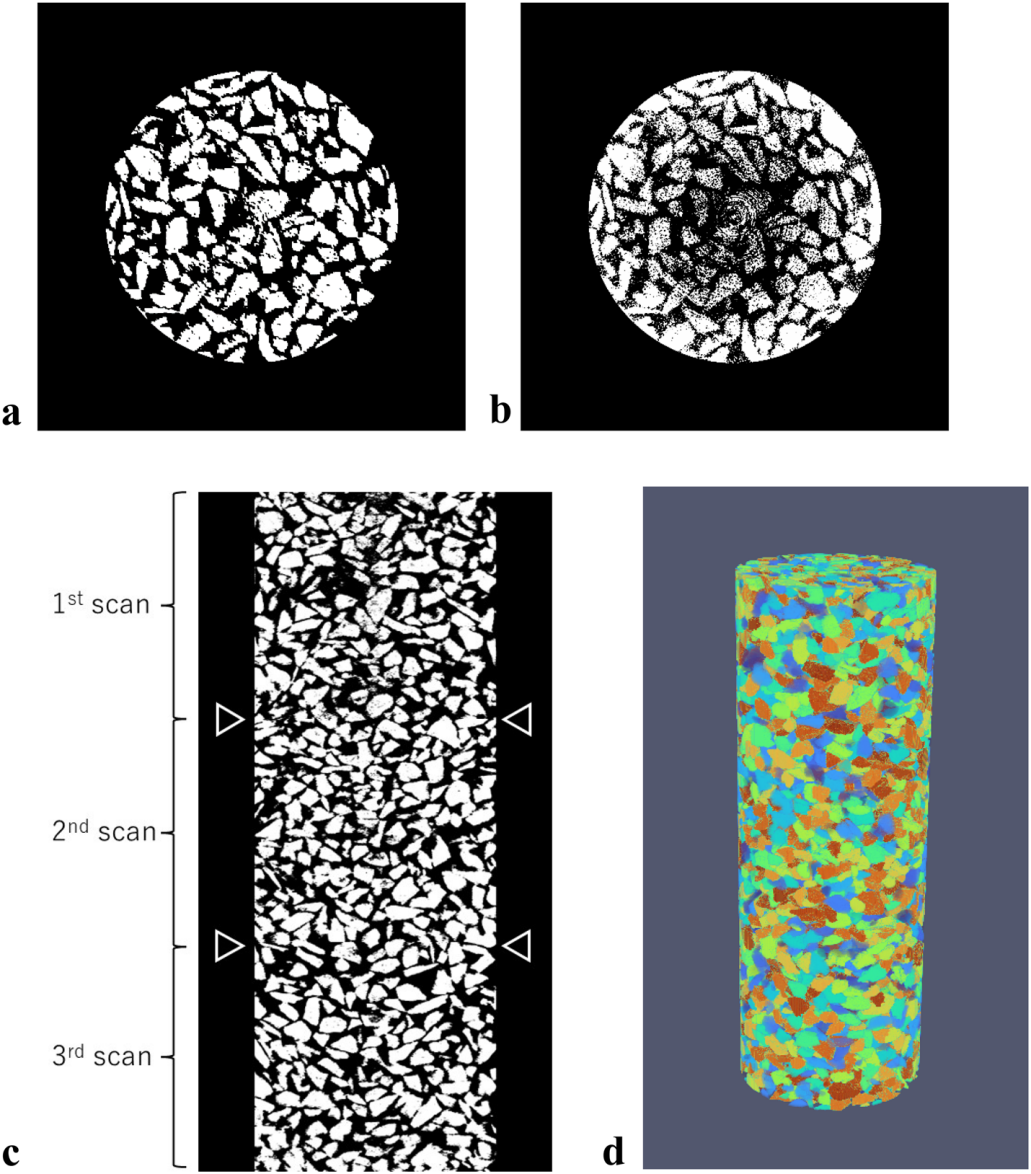
Results of image processing for the horizontal slice image: (**a**) after denoising and binarizing; (**b**) after binarizing without denoising; (**c**) after stitching (shown by vertical slice image); and (**d**) after segmenting (shown by different colors for segmented individual particles in 3D view).

Figure 13a shows an example of a horizontal slice image obtained using the first scan at the depth of 700 mm after denoising and binarizing as described in Section “Image processing and analysis for particle size and shape characteristics” (i.e., processing steps (i) and (ii)). As seen, these processes reasonably partition the particles. For comparison, Fig. 13b shows the image binarized by the same threshold value as that shown in Fig. 13a without denoising. Circles, that appear to be traces of the ring artifacts, were observed, and the scattering noise remained near the center. Figure 13c exhibits the vertical slice of the continuous soil sample images after the three different datasets of images were stitched as discussed in step (iii) of image processing in Section “Image processing and analysis for particle size and shape characteristics”. For every dataset, the particles could be reasonably binarized. Most importantly, no obvious gaps are seen around the two boundaries of different datasets indicated by the triangles, which implies that the particles were stable between the different scanning steps. The experimental results indicate that during drilling, the rotating drill blades excavate the soil, and the entire system advances vertically without skewing; thus, the soil sample retained in the sampling tube remains undisturbed. Figure 13d shows a 3D view after segmentation when individual particles are given different labels, i.e., consecutive numbers and colors, which corresponds to step (iv) of image processing. Finally, a digital soil sample, 56 mm in diameter and 155 mm in height, that included approximately 7000 irregularly shaped particles was generated.

**Figure 14:**
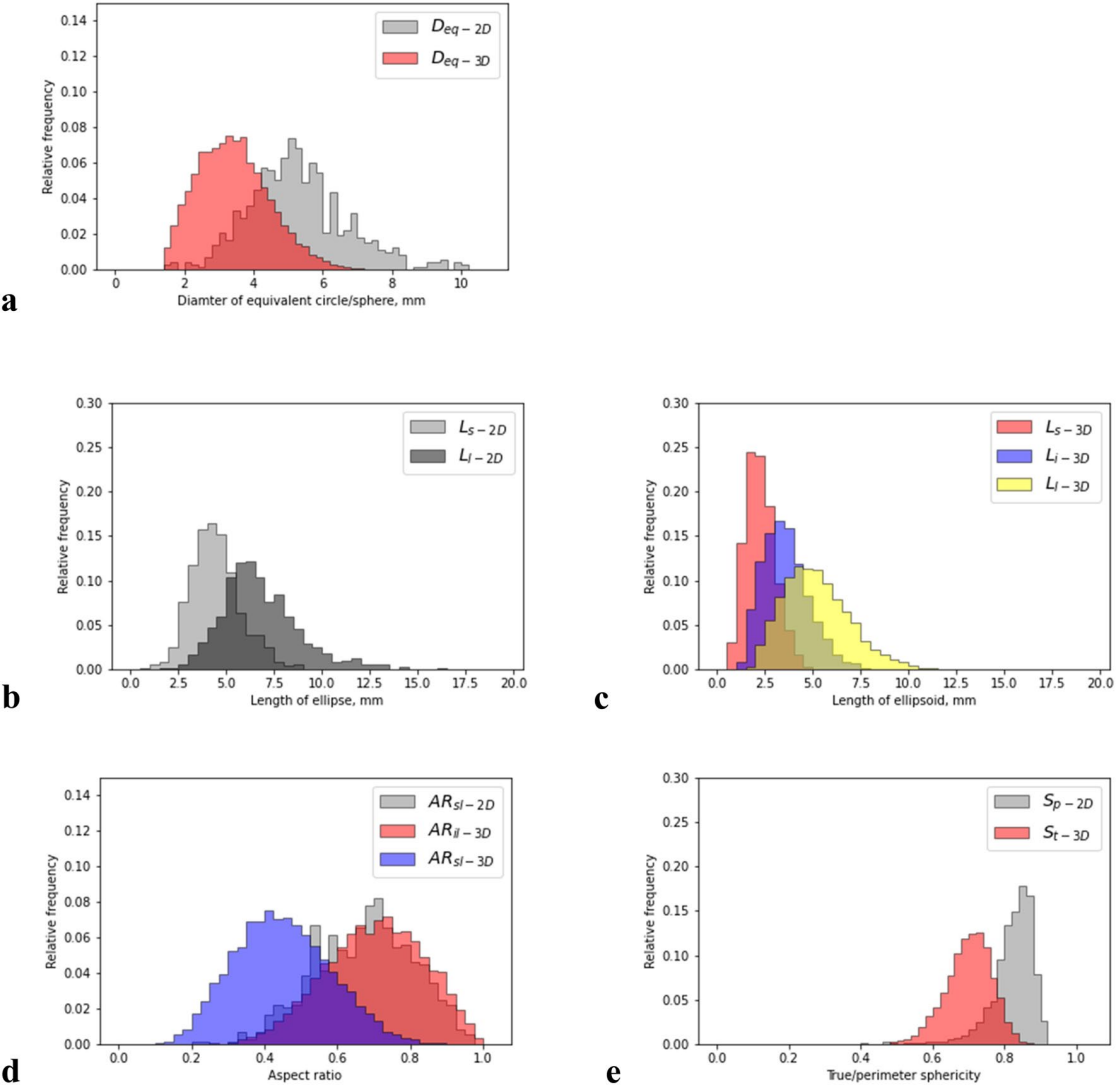
Comparison of particle size and shape characteristics; (**a**) diameters of equivalent circles/spheres; (**b**) length of the ellipse; (**c**) length of the ellipsoid; (**d**) aspect ratios; and (**e**) sphericities.

Figure 14 and Table 6 show the relative frequency distribution and results of the statistically analyzed particle size and shape characteristics, respectively. Comparing *D*_eq-2D_ and *D*_eq-3D_ in Fig. 14a, *D*_eq-2D_ reveals a distribution with larger diameters than those of *D*_eq-3D_. The mean value of *D*_eq-2D_ was 5.28 mm, whereas that of *D*_eq-3D_ was 3.42 mm. Because the mean grain size (*D*_50_) from sieve analysis was 4.1 mm (Table 3), these values spanned *D*_50_. As previously noted, the *D*_eq-2D_ is based on images of projected particles. In such a process, the shortest dimension of the particle is typically vertical and therefore not captured in the image where the projected dimensions are the largest and intermediate ones^49^. This can result in larger than actual estimates of particle sizes. In contrast, the *D*_eq-3D_ are based on actual consideration of all three dimensions of a particle (short, intermediate and long). This can result in smaller estimates than the corresponding *D*_eq-2D_ values. With mechanical sieving, the particles typically pass through based on the short and intermediate dimensions. As such, the actual size estimates may be smaller than the true estimates from CT studies. This tendency induced by the difference in dimensions based on the different procedures used to determine them also exists in the length parameters related to the morphologies of an ellipse (Fig. 14b) and ellipsoid (Fig. 14c). Therefore, comparing the lengths of the major axes showed that *L*_l-2D_ was slightly larger than *L*_l-3D_. *L*_s-2D_ was significantly larger than *L*_s-3D_ when comparing the lengths of the minor axes; this was probably because of the effect of difference of dimensions between 2 and 3D images as described before. In other words, because the shortest length of particles cannot be captured in 2D projected images when they are placed in a stable direction, *L*_s-2D_ is much closer to the intermediate length in 3D images, that is, *L*_i-3D_. Therefore, *L*_i-3D_ is distributed with a slightly lesser length than that of *L*_s-2D_, whereas their distribution profiles are reasonably similar. Accordingly, from Fig. 14d, *AR*_sl-2D_ corresponds to *AR*_il-3D_, but not to *AR*_sl-3D_. A similar tendency regarding the aspect ratios is also found in literature^16^. Comparisons of the sphericity in Fig. 14e and Table 6 show the differences in the mean values and histograms, where *S*_p-2D_ is 17% greater than *S*_t-3D_, and the histogram for *S*_p-2D_ tends to skew to the left. These differences are also indicated in Beemer et al.^16^; however, the best agreement between them was shown in Rorato et al.^14^. The differences may be attributed to the effect of pores in the particle regardless of whether they really exist or are created during the scanning and image processing as artifacts. Moreover, if the pores remain when *S*_t-3D_ is calculated, they decrease the volume of the particle, which affects the equivalent sphere volume, and increase its surface area, resulting in a decrease in *S*_t-3D_. Although the pores may somewhat similarly affect *S*_p-2D_, they can barely be captured in the 2D projected image. Ultimately, these results indicate that the dimensions of particles calculated using the 2D and 3D images approximately range within those from the sieving analysis, but they do not accord quantitatively. This is probably due to a dimensional difference; however, based on the aspect ratios, similar shape characteristics were observed in the 2D and 3D images.

**Table 6:** Statistical indicators for particle size and shape characteristics.

Items	Symbols	2D		3D	
		MV^a^	SD^b^	MV^a^	SD^b^
Diameter of the equivalent circle/sphere	*D*_eq-2D/3D_	5.28	1.44	3.42	1.03
Lengths of the ellipse/ellipsoid					
Minor axis	*L*_s-2D/3D_	4.45	1.29	2.24	0.76
Intermediate axis	*L*_i-3D_	–	–	3.56	1.19
Major axis	*L*_l-2D/3D_	6.77	2.02	5.19	1.72
Aspect ratios					
Minor/Major	*AR*_sl-2D/3D_	0.67	0.14	0.44	0.13
Intermediate/Major	*AR*_il-3D_	–	–	0.70	0.13
Perimeter/True sphericity	*S*_p-2D_/*S*_t-3D_	0.82	0.062	0.70	0.64

^a^Mean value.

^b^Standard deviation.

**Figure 15:**
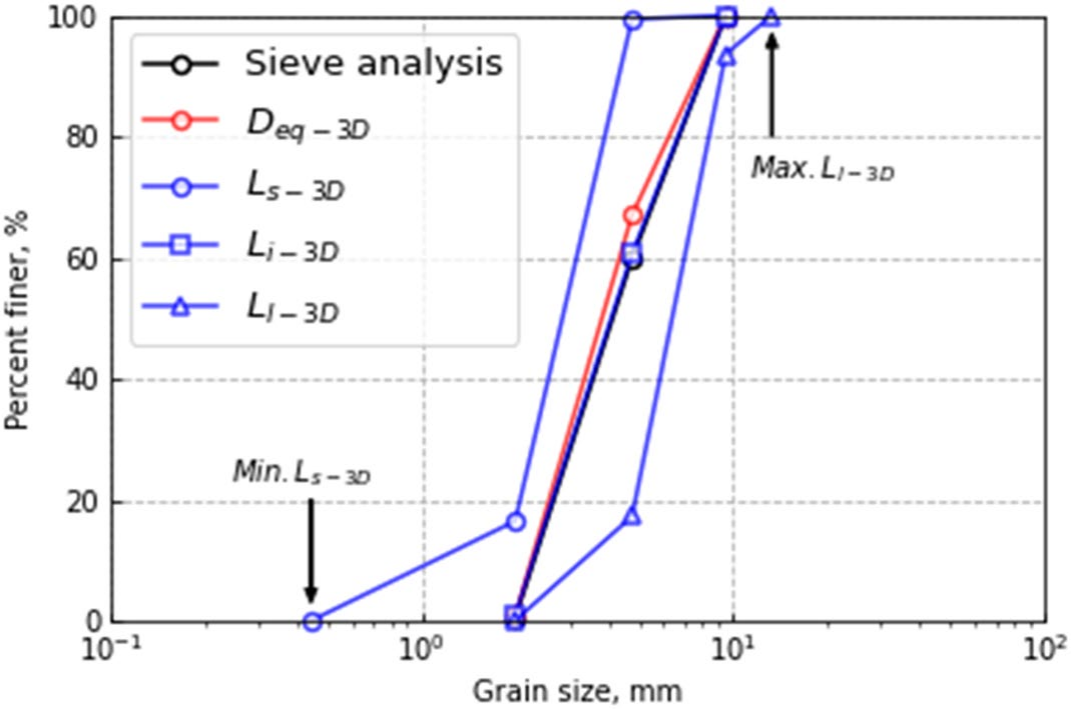
Comparison of GSD curves obtained from sieve and image analyses.

Figure 15 compares the GSD curves obtained by the sieve analysis and those calculated by the image analysis. For *D*_eq-3D_, the GSD curve is located slightly above that from the sieve analysis. This indicates that the calculated grain size is smaller than that obtained by the sieve analysis, which agrees well with the tendency shown in Fig. 14a. For the ellipsoid-fitted particles, while the GSD curves calculated by *L*_s-3D_ and *L*_l-3D_ are located on both sides of that obtained by the sieve analysis, that for *L*_i-3D_ appears to have the best agreement with it. These results imply that particles randomly pass through the sieves in various orientations with the lengths corresponding to *L*_s-3D_, *L*_i-3D_ or *L*_l-3D_, and eventually, the intermediate and average lengths i.e., *L*_i-3D_, determine the GSD curve.

### Discussion and future development needs

The preceding experiment and analysis results effectively confirmed that the soil samples retained in the sampling tube were scanned underground during drilling, and they were reconstituted to an equivalent digital soil sample based on the CT images. At this point, the feasibility of 3D-DIS using SeDX was considered to have been experimentally validated. However, to apply the technique in a real-world setting, several challenges associated with the coring, drilling, and scanning processes, which were not evaluated in detail in this study, should be addressed in future studies.

Although the image processing results confirmed that the drilling process did not disturb the soil sample retained in the sampling tube (Fig. 13c), the sampling tube (Section “Other equipment: sampling tube and drilling rig” and Fig. 3a) and the coring process including the refilling of the fresh gravel soil performed in the model test (Section “Testing processes”) cannot obtain intact soil samples, which have no or only slight disturbances during coring. The coring of intact soil samples requires a suitable method that matches the requirements of the target ground. Various methods have been proposed, standardized, and used for actual ground^1, 6, 35, 36^. Most of the sampling tubes are made of steel and unsuitable for scanning because their high specific gravity prevents the transmission of X-rays into the soil samples. Moreover, the diameter of the sampling tube used in this study is undersized for gravel soils, making it more difficult to acquire intact soil samples. Therefore, selecting suitable sampling tube materials and dimensions is critical.

Although the air-dried gravel soil was excavated to 800 mm in this study, for applications in actual subsurface conditions, drilling deeper into a fully or partially saturated ground, where SeDX may be exposed to higher earth pressures, will cause more challenges. In addition, a typical drilling process generates the flushing medium to remove cuttings, thus, lubricating or cooling the drilling tool will be important ^1^. Accordingly, the capacity for a higher drilling force along with waterproofing may further complicate the design of this equipment. Furthermore, the water in soil samples degrades the quality of CT images.

In this study, poorly-graded gravel soil was used, and the contained particles were successfully observed through CT scanning and image processing. The actual ground may consist of various sized particles. In such cases, smaller particles will not always be readily imaged because of the CT scanner’s limited resolution. Depending on the usage of the CT images, that is, whether particles with different sizes and shapes are required, or whether the global sedimentary structure must be captured, higher resolution-scanning and image-processing methods will be required.

Although the aforementioned challenges should be addressed, there are some promising applications. In this study, gravel soil was selected to verify the quality of acquired CT images through the image analysis of particle morphologies. However, if the objective of in-situ X-ray CT scanning is to evaluate a global dry density or void ratio of soil samples for construction control of embankment or ground improvement, for example, even sand, silt, or clay, which contain finer particles that cannot be visualized owing to a lack of resolution, can be a target ground. In this case, there could be more choices of sampling tubes^1^ that can be installed into SeDX. Moreover, although SeDX is expected to scan the soil samples underground, if scanning them on the ground surface is allowed, the compact and portable X-ray CT scanner (Fig. 2b) could be useful. In this case, it may be more effective in areas that are difficult for humans to access, such as a seabed ground.

## Conclusions

In this study, an in-situ X-ray CT scanning system that can drill into the ground and scan soil samples underground was developed. To investigate the feasibility of the equipment, a prototype model test was conducted using air-dried gravel soil. Furthermore, the images acquired in the model test were analyzed to verify whether the objects (i.e., soil particles) were visualized properly during the image analysis. The drilling system, with an installed X-ray CT scanner, successfully excavated the test ground to the desired depth (800 mm). A sampling tube was driven into the soil before drilling to protect the soil samples, which were scanned underground at depths of 700, 750, and 800 mm. Radiographs acquired during the test were utilized to generate the CT images. The image analysis results proved that CT images correctly visualized the size and shape characteristics of the gravel particles in the sampling tube and that the drilling process left the sedimentary structure of the samples undisturbed. The developments described in this manuscript represent a transformational technology that can advance subsurface characterization.

## Data Availability

The dataset generated during and/or analyzed during the current study is available from the corresponding author upon reasonable request.
